# siRNA/CS‐PLGA Nanoparticle System Targeting Knockdown Intestinal SOAT2 Reduced Intestinal Lipid Uptake and Alleviated Obesity

**DOI:** 10.1002/advs.202403442

**Published:** 2024-09-19

**Authors:** Jingjia Liang, Wentao Shao, Pu Ni, Qian Liu, Weirui Kong, Weiyi Shen, Qihan Wang, Anhua Huang, Guixin Zhang, Yulong Yang, Hongliang Xin, Zhaoyan Jiang, Aihua Gu

**Affiliations:** ^1^ State Key Laboratory of Reproductive Medicne and Offspring Health, School of Public Health Nanjing Medical University Nanjing 211166 China; ^2^ Key Laboratory of Modern Toxicology of Ministry of Education Center for Global Health Nanjing Medical University Nanjing 211166 China; ^3^ Collaborative Innovation Center for Cardiovascular Disease Translational Medicine Center for Global Health Nanjing Medical University Nanjing 211166 China; ^4^ Center of Gallstone Disease Shanghai East Hospital School of Medicine Tongji University Shanghai 201200 China; ^5^ School of Instrument Science and Engineering Southeast University Nanjing 210096 China; ^6^ General Surgery Department The Second Affiliated Hospital of Dalian Medical University Dalian 116027 China; ^7^ Department of Pharmaceutics School of Pharmacy Nanjing Medical University Nanjing 211166 China

**Keywords:** CD36, intestinal fatty acid absorption, obesity, PLGA‐b‐PEG, Sterol O‐acyltransferase 2

## Abstract

Effective inhibition of intestinal lipid uptake is an efficient strategy for the treatment of disorders related to lipid metabolism. Sterol O‐acyltransferase 2 (SOAT2) is responsible for the esterification of free cholesterol and fatty acids into cholesteryl esters. We found that intestine‐specific SOAT2 knockout (*Soat2^I‐KO^*) mice was capable to prevent the development of dietary induced obesity due to reduced intestinal lipid absorption. *Soat2* siRNA/CS‐PLGA nanoparticle system was constructed to enable intestinal delivery and inhibition of *Soat2*. This nanoparticle system was composed of PLGA‐block‐PEG and chitosan specifically delivering *Soat2* siRNAs into small intestines in mice, effectively inhibit intestinal lipid uptake and resolving obesity. In revealing the underlying mechanism by which intestinal SOAT2 regulating fatty acid uptake, enhanced CD36 ubiquitination degradation was found in enterocytes upon SOAT2 inhibition. Insufficient free cholesterol esterification promoted endoplasmic reticulum stress and recruitment of E3 ligase RNF5 to activate CD36 ubiquitination in SOAT2 knockdown enterocytes. This work demonstrates a potential modulatory function of intestinal SOAT2 on lipid uptake highlighting the therapeutic effect on obesity by targeting intestinal SOAT2, exhibiting promising translational relevance in the siRNA therapeutic–based treatment for obesity.

## Graphical abstract

Treatment with *Soat2* siRNA/CS‐PLGA nanoparticles prepared by CS‐PLGA nanoplatform is capable to inhibit SOAT2 in enterocytes. Intestinal SOAT2 inhibition leads to enhanced ubiquitination‐mediated degradation of intestinal CD36, a protein responsible for fatty acid uptake in enterocytes. E3 ligase RNF5 activated by endoplasmic reticulum stress is responsible for the ubiquitination degradation of CD36.

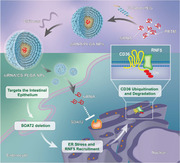

## 1 Introduction

During the recent decades, obesity is recognized as an epidemic metabolic disorder, entailing with dyslipidemia, hyperglycemia, insulin resistance, etc.^[^ ^1^ ^]^ Rise in high‐calorie food consumption coupled with decline in physical activity contribute to energy imbalance play pivotal roles leading to obese development.^[^ ^2^ ^]^ Limiting exogenous fat intake, inhibiting fatty acid synthesis and enhancing fatty acid degradation through β‐oxidation are suggested ways to prevent obese development. The gastrointestinal tract is the first gate of dietary fat intake. Various studies have tried to identify compounds targeting intestinal fatty acids absorption. Ezetimibe is a widely prescribed drug targeting the cholesterol transporter, Nieman Pick C1 like 1 (NPC1L1) protein, in the enterocyte to control cholesterol levels.^[^ ^3^ ^]^ With the reduction of 25% low‐density lipoprotein cholesterol (LDL‐C) levels,^[^ ^4^ ^]^ the combination treatment of ezetimibe/statins was shown to halt the progression of obesity‐related diseases as well, suggesting its role to limit intestinal fatty acid uptake. Although the molecular process of fatty acid uptake in enterocytes – membrane uptake, intracellular transportation and triglycerides assembly, has been elucidated, no specific inhibitor is proved to effectively prevent obese by inhibiting fatty acid absorption so far.

Sterol O‐acyltransferase 2 (SOAT2, also known as acyl‐CoA: cholesterol acyltransferase, ACAT2 previously) is an enzyme to esterified free cholesterol and fatty acids into cholesteryl esters (CE),^[^ ^5^, ^6^ ^]^ preferring to use oleate and palmitate acids as substrates. Due to its unique presence in hepatocytes and enterocytes and role in very low‐density lipoprotein/chylomicron‐CE synthesis, its role in association with the development of atherosclerosis has been extensively studied. Especially, inhibition of hepatic SOAT2 was shown to prevent atherosclerosis development.^[^ ^7^, ^8^ ^]^ Due to its known role on CE production and much concerns on atherosclerosis, the role of SOAT2 on regulating fatty acid metabolism is somehow neglected with only concerns by the fact that hepatic inhibition of SOAT2 unexpectedly resulting in increase of triglycerides accumulation.^[^ ^9^, ^10^ ^]^ In SOAT2 whole knockout mice fed with high fat diet (HFD), hepatic triglycerides accumulation and insulin resistance were ameliorated.^[^ ^11^ ^]^ Though limited, these evidences suggested the role of intestinal SOAT2 in controlling dietary fatty acid absorption, which gave a hint that intestinal SOAT would be a promising target to modulate the incidence of diet induced obesity.

The first approval of the small interfering RNA (siRNA)‐based drug (patisiran) by the U.S. Food and Drug Administration (FDA) for treating hereditary transthyroxine protein‐mediated (hATTR) amyloidosis brought into a generation of nanocarrier‐assisted siRNA therapy epoch.^[^ ^12^, ^13^ ^]^ Nanoparticles are vehicles to augment the efficacy for drug delivery and therapy. Poly (lactic‐co‐glycolic acid) (PLGA) nanoparticles have been used as appropriate carriers for delivering siRNA in colorectal cancer therapy. Cells can internalize PLGA nanoparticles (NPs), enabling the release of functional siRNA into the cytoplasm. Coating nanoparticles with high‐affinity molecules, like antibodies, enables them to target aberrantly expressed molecules on the cell surface.^[^ ^14^ ^]^ The positive electrical properties of chitosan (CS), a coated material, facilitate the attraction of the nanosystem to the negatively charged intestinal mucosa, enabling effective siRNA delivery to the intestine.^[^ ^15^ ^]^ Due to its efficacy in siRNA delivery and cellular targeting, a novel PLGA‐siRNA coated with CS targeting SOAT2 in enterocytes would be a promising therapeutic option to modulate intestinal fatty acid uptake to prevent obese.

In this study, we constructed a novel nanoparticle system comprising chitosan to deliver *Soat2* siRNAs within the intestine. The nano‐formulated, *Soat2* siRNA/CS‐PLGA nanoparticles, could be attracted by the negatively charged intestinal mucosa through the positive charge of CS, enabling its retention in the intestine and facilitating the delivery of siRNA to the intestinal epithelium. Treatment with *Soat2* siRNA/CS‐PLGA nanoparticles was capable to inhibit SOAT2 in enterocytes, which would be adequate to prevent the development of high‐fat diet induced obesity. We further demonstrated that the therapeutic effect of *Soat2* siRNA inhibition to limit fatty acid uptake in enterocytes was due to an enhancement of ubiquitination associated degradation of CD36, a fatty acid transporter in the enterocyte.

## 2 Results

### 2.1 Intestinal Loss of SOAT2 Prevented Diet Induced Obesity by Decreasing Fatty Acid Absorption

**Figure 1:**
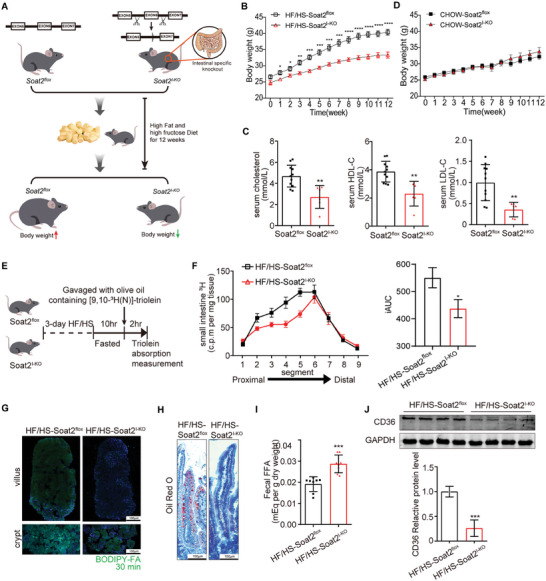
Loss of SOAT2 in intestine prevented diet induced obesity by decreasing intestinal lipid absorption. A) Scheme of experimental procedure. B) Weight gain of *Soat2* ^*I‐KO*^ mice measured at each week of HF/HS diet (n = 12). C) Serum cholesterol levels in *Soat2* ^*I‐KO*^ mice on HF/HS diet. D) Body weight of *Soat2* ^*I‐KO*^ mice measured at each week on chow diet. E,F) Distribution of ^3^H‐labeled fatty acid signals and the integrated area under the curve (iAUC) absorbed in each segment of small intestine from *Soat2* ^*I‐KO*^ or wild‐type (*Soat2* ^*Flox*^) mice after oral gavage. G) *Ex vivo* evaluation of fatty acid absorption in small intestine from *Soat2* ^*I‐KO*^ or wild‐type (*Soat2* ^*Flox*^) mice. Crypt and villus epithelial cells were isolated from the proximal half of the small intestine from mice and incubated with BODIPY^TM^ FL C_16_ (fluorescent palmitic acid) and observed under confocal microscope. H) Oil red O staining of villus of proximal small intestine from fast and re‐fed *Soat2* ^*I‐KO*^ or wild‐type (*Soat2* ^*Flox*^) mice after 3‐day high fat diet. I) Quantification of free fatty acid levels in feces from *Soat2* ^*I‐KO*^ or wild‐type (*Soat2* ^*Flox*^) mice. J) Protein expression of CD36 protein in the proximal small intestines from *Soat2* ^*I‐KO*^ or wild‐type (*Soat2* ^*Flox*^) mice by Western Blot. Data were expressed as means SEM. “*” indicated *p* < 0.05, “**” indicated *p* < 0.01, “***” indicated *p* < 0.001, and “****” indicated *p* < 0.0001.

To observe the unique role of intestinal SOAT2 on obese, the intestinal specific *Soat2* knockout (*Soat2^I‐KO^*) mice were fed with high fat and high sugar diet (HF/HS) for 12 weeks (**Figure** **1**A). The *Soat2^I‐KO^* mice exhibited apparently less weight gain (Figure 1B) than their wild‐type (WT) littermates. *Soat2^I‐KO^* mice exhibited lower body weight, smaller body sizes (Figure S1, Supporting Information). Inguinal WAT fat pads and adipocyte size were smaller in the *Soat2^I‐KO^* mice than in the WT littermates (Figure S2, Supporting Information). The difference was not due to changes in daily food intake (Figure S3, Supporting Information). Serum cholesterol, high‐density lipoproteincholesterol (HDL‐C) and LDL‐C was significantly decreased in the *Soat2^I‐KO^* mice (Figure 1C). These data indicated that loss of *Soat2* in intestine was enough to protect mice from the development of HF/HS‐induced obesity. In contrast, no difference in body weight gain was observed in the *Soat2^I‐KO^* mice fed with chow diet compared with their wild‐type (WT) littermates (Figure 1D).

Measuring the efficiency of fatty acid uptake in the whole small intestine of *Soat2^I‐KO^* mice, we found decreased [^3^H]‐fatty acid signals in the proximal 2/3 part of the small intestine of *Soat2^I‐KO^* mice (Figure 1E,F). Villus epithelial cells were then isolated from proximal small intestine of *Soat2^I‐KO^* mice and incubated with fluorescent palmitic acid. More slowly and less labeled fatty acids absorption was observed in epithelial cells from *Soat2^I‐KO^* mice (Figure 1G). Fast‐refed fatty acid experiment also demonstrated less lipid accumulation in the villus of proximal small intestine from *Soat2^I‐KO^* mice (Figure 1H). As expected, increased fecal excretion of free fatty acids was found in *Soat2^I‐KO^* mice (Figure 1I). Both levels of triglyceride and total cholesterol decreased in the proximal small intestine of *Soat2^I‐KO^* mice (Figure S4, Supporting Information). No difference in the whole intestine length was found between *Soat2^I‐KO^* mice and WT littermates (Figure S5, Supporting Information). Not any pathological damage was found in intestine, either (Figure S5, Supporting Information).

The above data indicated a role of intestinal SOAT2 inhibition to control intestinal fatty acids uptake. We then measured the expression of known genes involved in cellular fatty acid transportation, but found no change at the mRNA or protein level (Figure S6, Supporting Information). Very interestingly, we found significantly decrease of CD36 protein, a recognized protein in fatty acids uptake,^[^ ^16^ ^]^ in *Soat2^I‐KO^* mice (Figure 1J). These results designated the fact that decrease of fatty acids absorption when loss of intestinal SOAT2 was due to decrease of CD36 protein.

### 2.2 Preparation and Characterization of CS‐PLGA Nanoparticles

**Figure 2:**
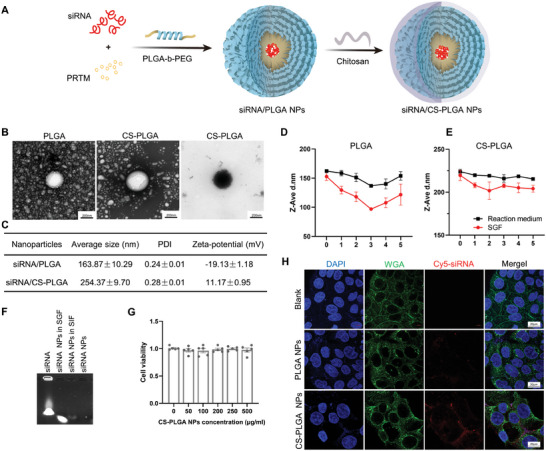
Preparation and characterization of siRNA/CS‐PLGA nanoparticles system. A) The reaction scheme of nanoparticle synthesis. B) Representative transmission electronic microscopy images of PLGA (scale bars: 100 nm) and CS‐PLGA nanoparticles (left). C) Zeta‐potential of PLGA and CS‐PLGA nanoparticles. Size distribution of CS‐PLGA nanoparticles measured by Mastersizer Micro. D–F) Stability of CS‐PLGA nanoparticles in the simulated gastric fluid (SGF) and simulated intestinal fluid (SIF) for the indicated times (n = 3 / group). G) Cell viability of CS‐PLGA nanoparticles treatment with different concentrations. H) Absorption of Cy5‐siRNA by cells under treatment with PLGA or CS‐PLGA nanoparticles.

We next designed an oral siRNA‐based nanoparticle system using PLGA and CS based on the pH‐sensitive and adhesive effect of CS to specifically knock‐down *Soat2* in the enterocytes. PLGA‐COOH with carboxyl group was first activated by N,N'‐Dicyclohexylcarbodiimide (DCC) and N‐Hydroxy succinimide (NHS) in dichloromethane, and then coupled with the biffunctional group polyethylene glycol (PEG) to synthesize poly(D,L‐lactic‐co‐glycolic acid)‐block‐polyethylene glycol (PLGA‐b‐PEG). Degradable CS‐PLGA nanoparticles were prepared using PLGA‐b‐PEG polymer (as the core) coated with CS shell (as a protective layer against stomach acid) (**Figure** **2**A). The CS‐PLGA nanoparticles were roughly spherical in shape and had a particle diameter of ≈254 nm, as analyzed by TEM and light scattering spectrometry (Figure 2B). The polymer disparity index (PDI) and zeta potential were 0.28 ± 0.01 and 11.17 ± 0.95 mV, respectively (Figure 2C). Essential to the nanoparticle system, the protonation of the CS shell stabilized the PLGA‐b‐PEG nanoparticles under acidic conditions. In contrast, the shell collapsed in the simulated intestinal fluid (SIF), an in vitro system mimicking the physical environment in the small intestines (Figure 2D–F). Then, Caco2 cells were treated with CS‐PLGA nanoparticles carrying Cy5‐siRNA (with red fluorescence) for 4  h. The CS‐PLGA nanoparticle‐coated siRNA was more prone to be absorbed than the PLGA nanoparticles (Figure 2G,H), due to the presence of stronger positive electricity of the CS‐PLGA nanoparticles.

**Figure 3:**
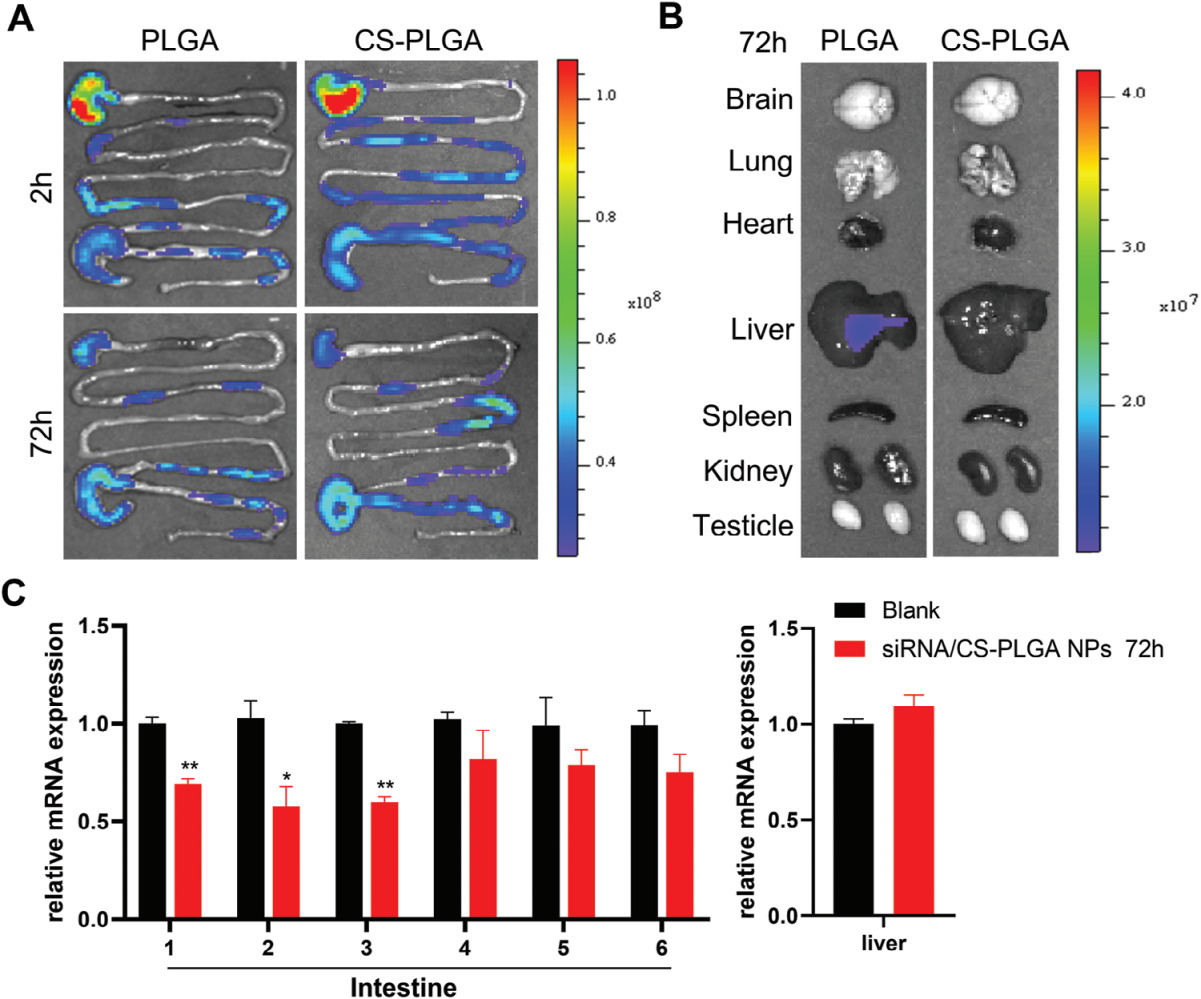
Distribution of CS‐PLGA nanoparticles in vivo. A) Representative images of the intestinal tract of mice during 72 h after administration of either Cy5 siRNA/PLGA or Cy5 siRNA/CS‐PLGA nanoparticles by intragastric administration. B) Biodistribution of Cy5 siRNA/PLGA and Cy5 siRNA/CS‐PLGA nanoparticles for 72 h in the mouse brain, lung, heart, liver, spleen, kidney and testicle. C) The Soat2 RNA expression in each segment of small intestine after administration of Soat2 siRNA/CS‐PLGA nanoparticles for 72 h. Quantitative data were presented as mean ± SEM. “*” indicated *p* < 0.05, “**” indicated *p* < 0.01.

To assess the biological distribution of CS‐PLGA nanoparticles, Cy5‐siRNA was loaded into PLGA to form fluorescent nanoparticles for in vivo tracking. The mice were given Cy5‐siRNA/CS‐PLGA nanoparticles (40 µg kg^−1^) and equivalent Cy5‐siRNA/PLGA nanoparticles by gavage for 72 h. Compared with Cy5‐siRNA/PLGA nanoparticles, Cy5‐siRNA/CS‐PLGA nanoparticles accumulated exclusively within the small intestine and sustained till 72 h (**Figure** **3**A). No fluorescence signal was observed in other metabolic organs, including the brain, heart, lungs, kidney, spleen or testicle (Figure 3B). The inhibitory effects on Soat2 mRNA expression of each mouse intestinal segment were measured at different time points after siRNA/CS‐PLGA NP treatment. The potent effects were observed mainly in the proximal part of the small intestine (segment 1–3), but not the distal parts (segment 5–6). The effect existed until 72 h after treatment (Figure 3C; Figure S7A, Supporting Information). No inhibitory effect on hepatic Soat2 expression was observed during the period of treatment (Figure S7B, Supporting Information).

### 2.3 *Soat2* siRNA/CS PLGA Nanoparticles Ameliorated Obesity Development in Mice

**Figure 4:**
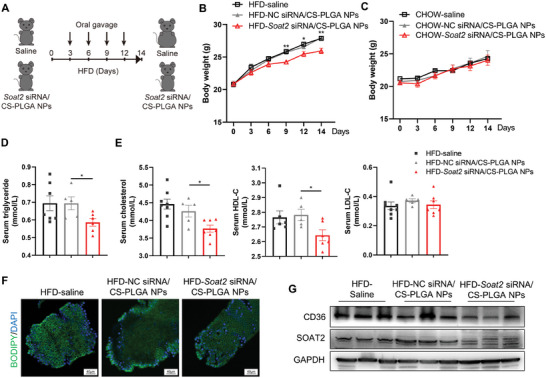
Therapeutic effect of *Soat2* siRNA/CS‐PLGA nanoparticles in mice. A) Experimental scheme. B,C) Body weight of mice treated with *Soat2* siRNA/CS‐PLGA nanoparticles measured at each 3 days on high fat diet or chow diet (n = 6). D,E) Quantification of serum lipid levels. F) Decreased intestinal fatty acid uptake in mice treated with *Soat2* siRNA/CS‐PLGA nanoparticles measured by *ex vivo* quantification of fatty acid absorption in small intestine with BODIPY^TM^ FL C_16_ (fluorescent palmitic acid). G) Protein expression of CD36 protein in the intestines from mice treated with *Soat2* siRNA/CS‐PLGA nanoparticles by Western Blot. Quantitative data were presented as mean ± SEM. “*” indicated *p* < 0.05, “**” indicated *p* < 0.01.

To test the therapeutic effect of *Soat2* siRNA/CS‐PLGA nanoparticles, male mice fed a high‐fat diet (HFD) were given *Soat2* siRNA/CS‐PLGA nanoparticles every 3 days (**Figure** **4**A). *Soat2* siRNA/CS‐PLGA nanoparticles treatment led to less weight gain than control mice with HFD (Figure 4B) in line with that observed in *Soat2^I‐KO^* mice. No difference in body weight gain was observed in mice treated with *Soat2* siRNA/CS‐PLGA nanoparticles fed with chow diet (Figure 4C). Administration of *Soat2* siRNA/CS‐PLGA nanoparticles significantly reduced serum triglycerides and cholesterol levels (Figure 4D,E). Crypt epithelial cells and villous epithelial cells isolated from the proximal small intestine of mice were incubated with fluorescent palmitic acid (BODIPY^TM^ FL C_16_). The results confirmed slower and less labeled fatty acid absorption in *Soat2* siRNA/CS‐PLGA nanoparticles treated mice (Figure 4F). In addition, the intestinal SOAT2 and CD36 proteins reduced in mice treated with *Soat2* siRNA/CS‐PLGA nanoparticles (Figure 4G).

**Figure 5:**
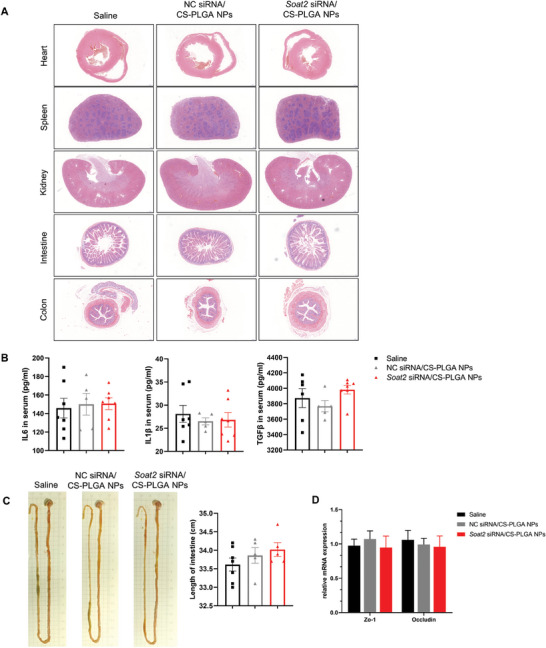
Evaluation of safety of *Soat2* siRNA/CS‐PLGA nanoparticles in vivo. A) The HE staining of tissues including heart, spleen, kidney, small intestine and colon (n = 6). B) Serum immune‐related cytokine determined by ELISA assays. C) The length of the intestine. D) qRT‐PCR measurements of intestinal barrier proteins in mice treated with *Soat2* siRNA/CS‐PLGA nanoparticles. Quantitative data were presented as mean ± SEM. “*” indicated *p* < 0.05, “**” indicated *p* < 0.01.

The safety of CS‐PLGA nanoparticles was evaluated in Caco2 cells. None of the nanoparticles used affected the viability of cells (Figure 2F), suggesting that CS‐PLGA nanoparticle was safe for intestinal cells. Moreover, in mice gavaged with *Soat2* siRNA/CS‐PLGA nanoparticles, no pathological damage to various organs was observed (**Figure** **5**A). No inflammatory reaction was observed as measured by serum cytokine assay (Figure 5B). There was no difference in the length of the small intestine and colon between the two groups (Figure 5C). The results of qRT‐PCR also showed no injury in the intestinal barrier proteins of mice treated with *Soat2* siRNA/CS‐PLGA nanoparticles (Figure 5D). These results collectively confirmed the safety of *Soat2* siRNA/CS‐PLGA nanoparticles for in vivo application.

### 2.4 SOAT2 Deficiency Promoted RNF5‐Mediated Ubiquitination and Degradation of CD36

**Figure 6:**
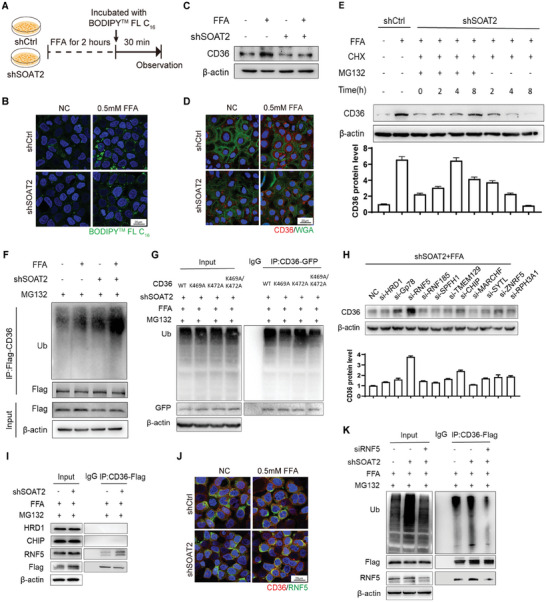
Decreased fatty acid absorption by RNF5‐mediated ubiquitination degradation of CD36 in SOAT2 knockdown (shSOAT2) Caco2 cells. A) Flow chart showing the measurement of fatty acid absorption in Caco2 cells using BODIPY^TM^ FL C_16_. B) Decreased absorption capacity of fatty acid in the shSOAT2 Caco2 cells. C) CD36 protein expression in the shSOAT2 Caco2 cells. D) Immunofluorescence staining of CD36 protein in the shSOAT2 Caco2 cells. E) Representative WB images of CD36 protein levels after SOAT2 knockdown combined with 100 µg mL^−1^ Cycloheximide (CHX, a protein synthesis inhibitor) and MG132 (10 µm) treatment in indicated time duration. F) CD36 ubiquitination was determined in Caco2 cell by IP of anti‐Flag followed by WB with anti‐Ubiquitin (Ub) after transfecting with CD36‐flag. shSOAT2 or control Caco2 cells were treated with/without 0.5 µm FFA. After 18 h, cells were treated with 10 µm MG132 for another 6 h. G) The Ub was determined by IP/WB after transfection with GFP‐CD36 (WT, K469A or K472A) in the presence of MG132 (10 µm) in shSOAT2 Caco2 cells. H) Representative WB images of CD36 in shSOAT2 Caco2 cells after knockdown by a panel of ER‐resident E3 ligases (HRD1, Gp78, RNF5, RNF185, SPFH1, TMEM129, CHIP, MARCHF, SYTL, ZNRF5, RPH3A1). I) The interaction of RNF5, HRD1 and CHIP with CD36 were detected by IP of anti‐Flag‐CD36 followed by WB in shSOAT2 Caco2 cells. J) Immunostaining of co‐localization of RNF5 and CD36 in Caco2 cells. K) The CD36 ubiquitination was determined by IP/WB after RNF5 knockdown in the presence of MG132 in shSOAT2 Caco2 cells. All data are derived from three independent experiments and the quantification data were presented as mean ± SEM in the bar graph. Values in the control groups were normalized to 1.

In order to explore the mechanism how intestinal SOAT2 deficiency affected fatty acids absorption in enterocytes, a SOAT2 knockdown Caco2 (shSOAT2) cell was constructed as an enterocyte model. These cells exhibited apparent decrease of BODIPY^TM^ FL C_16_‐labeled fatty acid uptake (**Figure** **6**A,B), presenting a reproducible in vitro phenotype on intestinal fatty acid uptake as *Soat2* siRNA/CS‐PLGA nanoparticle treated mice and *Soat2^I‐KO^* mice.

In control cells, free fatty acids incubation induced CD36 protein levels (Figure 6C). However, in the shSOAT2 cells, CD36 protein could not be up‐regulated (Figure 6C). This was not due to any changes in subcellular localization of CD36 (Figure 6D). Since CD36 could be rapidly degraded in response to fatty acid concentration,^[^ ^17^, ^18^ ^]^ we explored homeostasis regulation of CD36 in the shSOAT2 Caco2 cells. The protein stability was first analyzed by cycloheximide (CHX, a protein synthesis inhibitor) chase assay. CD36 protein levels decreased time‐dependently in the control group, but not in MG132 (a potent proteosome inhibitor) treated group (Figure 6E). The data indicated that endogenous CD36 proteins were prone to be degraded through the proteasome pathway.

Enrichment of ubiquitination of CD36 protein was found in the shSOAT2 cells (Figure 6F). Sequence analysis and proteomic database search indicated presence of two highly conserved lysine clusters, K472 and K469, being the potential ubiquitination sites for CD36 (Figure S8A, Supporting Information). To differentiating the ubiquitination site, we generated CD36 mutants (single K472R or K469R site and double sites) by producing lysine to arginine mutations. Decreased ubiquitination level of CD36 protein was observed when K469 was mutated, but not K472 (Figure 6G).

The potential E3 ligases involved in CD36 protein ubiquitination were predicted using UbiBrowser (MARCHF, SYTL, ZNRF5, and RPH3A1A, Figure S8B, Supporting Information). Since SOAT2 is located at endoplasmic reticulum (ER), we constructed an siRNA library for screening CD36 ubiquitination binding E3 ligases selected from those predicted ligases with ER localization (Gp78, RNF5, RNF185, HRD1, SPFH1, CHIP, and TMEM129). The results showed that the level of CD36 protein increased significantly only when RNF5 was knockdown (Figure 6H). The results of immune‐precipitation (IP) experiments demonstrated an interaction of CD36 with RNF5 (Figure 6I). The fluorescence imaging of cells also supported co‐localization of CD36 and RNF5 (Figure 6J). To further investigate the regulation of RNF5 on CD36 protein stability, we knock‐down RNF5 in the shSOAT2 cells. Loss of RNF5 resulted in a significant decrease in CD36 ubiquitination, accompanied by an increase in CD36 protein abundance (Figure 6K). Overall, these results demonstrated that RNF5 was the key E3 ligase for ubiquitination and degradation of CD36 when SOAT2 was knockdown in enterocytes.

### 2.5 ER Stress in SOAT2 Knockdown Cells Promoted RNF5‐Mediated ER‐Related Degradation

**Figure 7:**
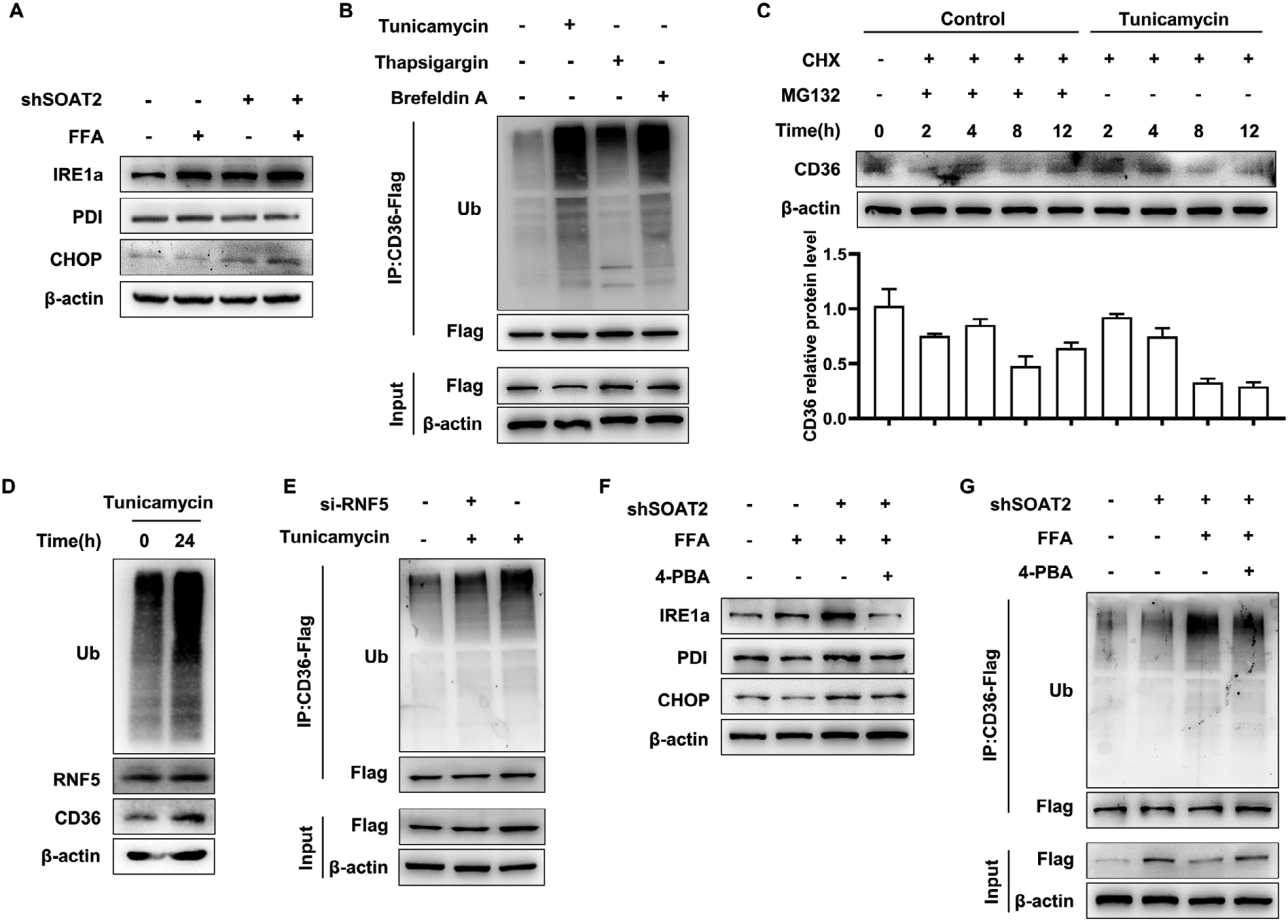
ER stress in SOAT2 knockdown (shSOAT2) Caco2 cells promote RNF5‐mediated ER‐related degradation. A) Expression of endoplasmic reticulum stress‐related proteins in the shSOAT2 Caco2 cells. B) The CD36 ubiquitination was determined by IP/Western Blot after tunicamycin, brefeldin A or thapsigargin treatment in the presence of MG132. C) Representative WB images of CD36 protein levels after tunicamycin treatment combined with 100 µg mL^−1^ CHX (protein synthesis inhibitor) and MG132 (10 µm) treatment in indicated time duration. D) The ubiquitination of whole cell was determined by IP/Western blot after tunicamycin treatment in the presence of MG132. E) The CD36 ubiquitination was determined by IP/WB after RNF5 knockdown in the presence of MG132 in cells treated with tunicamycin. F) Expression of endoplasmic reticulum stress‐related proteins in the shSOAT2 Caco2 cells after 4‐PBA treatment. G) The CD36 ubiquitination was determined by IP/Western Blot after 4‐PBA treatment in the presence of MG132 in the shSOAT2 Caco2 cells. All data are derived from three independent experiments and the quantification data were presented as mean ± SEM in the bar graph. Values in the control groups were normalized to 1.

As an E3 ligase, RNF5 is involved in ER‐associated degradation (ERAD) in response to ER stress. The ER stress related protein, CHOP and IRE1α, increased in the shSOAT2 cells and in the proximal intestine of *Soat2^I‐KO^* mice (**Figure** **7A**; Figure S9, Supporting Information). Caco2 cells were treated with well‐established ER stress inducers (brefeldin A, thapsigargin and tunicamycin. All three ER stress inducers markedly promoted CD36 ubiquitination (Figure 7B). Among them, tunicamycin treatment promoted the most pronounced decrease in CD36 levels. Co‐incubation of cells with MG132 and cycloheximide (CHX attenuated tunicamycin effects, suggesting that CD36 undergoes proteasome‐dependent degradation in response to ER stress (Figure 7C). Especially, tunicamycin‐induced CD36 ubiquitination decreased when RNF was knockdown (Figure 7D,E).

To elucidate the important role of SOAT2‐dependent ER stress in RNF5‐mediated ER related degradation, we used 4‐phenylbutyric acid (4‐PBA) to alleviate ER response on the basis of SOAT2 knockdown cells. 4‐PBA is a molecular chaperone that assists in post‐transcriptional modification and folding of proteins in the ER, thus alleviating non‐folded protein reactions. 4‐PBA reduced the protein level of IRE1α, BIP and CHOP (Figure 7F). FFA or shSOAT2 could increase the level of IRE1α, and 4‐PBA could reduce the enhanced level of IRE1α induced by FFA or shSOAT2 alone (Figure S10, Supporting Information). When the ER stress was inhibited, the ubiquitination of CD36 protein reduced significantly (Figure 7G). This data indicated the involvement of ER stress to evoke the RNF5‐mediated degradation of CD36 protein upon SOAT2 knockdown.

### 2.6 RNF5‐Dependent Downregulation of CD36 in SOAT2 Knockdown Cells is Associated with Cholesterol‐Dependent Protein Misfolding

SOAT2 knockdown altered cellular free cholesterol levels (Figure S11A, Supporting Information). The fluctuation of free cholesterol level in ER regulated by SOAT2 may be a trigger of ER stress. Ezetimibe is a potent and selective cholesterol absorption inhibitor targeting membrane cholesterol transporter NPC1L1. In contrast to SOAT2 knockdown, ER stress was mitigated in shSOAT2 cells treated with ezetimibe (Figure S11B, Supporting Information), consequently following with the ubiquitination and degradation of CD36 proteins (Figure S11C, Supporting Information). This suggested the dependence of ER stress to promote ubiquitination and degradation of CD36 when SOAT2 was knockdown.

## 3 Discussion

**Figure 8:**
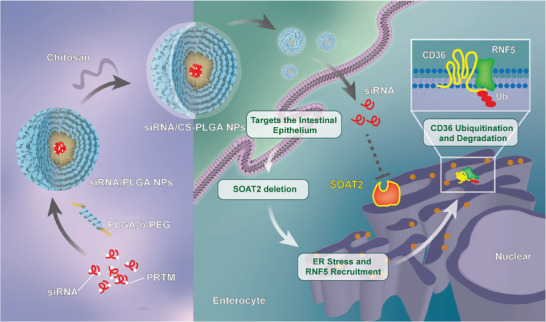
Schematic illustration of the preparation of CS‐PLGA nanoplatform and its principle of protecting efficacy by delivering *Soat2* siRNA. The nano‐formulated, *Soat2* siRNA/CS‐PLGA nanoparticles, could be attracted by the negatively charged intestinal mucosa through the positive charge of chitosan (CS), enabling its retention in the intestine and facilitating the delivery of siRNA to the intestinal epithelium. *Soat2* siRNA/CS‐PLGA nanoparticles is capable to inhibit SOAT2 in enterocytes. Intestinal SOAT2 inhibition leads to enhanced ubiquitination‐mediated degradation of intestinal CD36, a protein responsible for fatty acid uptake in enterocytes. ERAD‐related E3 ligase RNF5 activated by endoplasmic reticulum stress is responsible for the ubiquitination degradation of CD36.

Our study developed PLGA‐b‐PEG and CS coupled *Soat2* siRNA nanoparticle system targeting inhibition of intestinal SOAT2 to control dietary fatty acid intake so as to prevent the development of dietary induced obesity. We also identified a novel mechanism that intestinal SOAT2 regulated fatty acid intake through enhancing ER stress‐related ubiquitination degradation of CD36 in enterocytes (**Figure** **8**).

Up to now, several drugs have been developed for the treatment of obesity, mainly by direct targeting appetite inhibition such as sympathomimetics, serotonin 2C receptor agonists, opioid antagonists, dopamine‐norepinephrine reuptake inhibitors, and glucagon‐like peptide‐1 receptor agonists.^[^ ^19^ ^]^ Some have been withdrawn from the market due to concerns about cardiovascular safety^[^ ^20^ ^]^ and unexpected psychiatric side effects.^[^ ^21^ ^]^ Other drugs that promote weight loss by inhibiting pancreatic lipase to inhibit the absorption of dietary fat, although safety is very good, but the efficacy is somewhat moderate.^[^ ^22^ ^]^ Our study constructed a new promising intestinal specific inhibitor, the *Soat2* siRNA/CS‐PLGA nanoparticles, with great potential to treat obesity and obesity‐related metabolic disorders by inhibiting dietary fatty acid uptake from intestine.

The constructed nanoparticle system of *Soat2* siRNA in this study was able to deliver siRNA to and only deposited in the intestinal epithelial cells, without further entering into the blood. The cell targeting and uptake efficacy of nanoparticles are enhanced by PLGA coating CS since the wrapping of CS acts as a protective shelter to avoid degradation by stomach acids and retain functional siRNA until reaching small intestine. Since CS has a positive surface charge, it can safely interact with intestinal mucosal proteins. Therefore, CS‐PLGA nanoparticles can be more effectively absorbed within intestinal cells.^[^ ^15^ ^]^ The other important advantage of our nanosystem of *Soat2* siRNA is that the nanoparticles would not enter the circulation system, preventing its inhibitory effect on hepatic SOAT2 activity that might adversely lead to accumulation of triglycerides^[^ ^9^, ^10^ ^]^ The *Soat2* siRNA/CS‐PLGA nanoparticles have good biocompatibility to withstand digestion of gastrointestinal digestive fluid, specifically and effectively silence intestinal target genes. Moreover, CS‐PLGA nanoparticles are safe since no observable local immune response in the gut or other key organs were detected. In comparison, the commonly used adeno‐associated viruses (AAV) technique has difficulty to achieve high titer AAV, limiting its application. Furthermore, our CS‐PLGA nanoparticles system showed efficient inhibition of SOAT2 till 72 h after intake. Thus, CS‐PLGA nanoparticles represent a safe and effective system to be intestine specific targeting.

Most of the SOAT2 inhibitors to date cannot be used in clinical practice so far because of their low effect and difficulty in preparation. The SOAT2 inhibitor pyripyropene A, a fungal extract, has also been found to reduce cholesterol by inhibition of SOAT2 in primates, but cannot be produced in large quantities, due to the complex preparation process.^[^ ^23^ ^]^ The CS‐PLGA nanosystem we used could be easily constructed and prepared in large quantities. They had the advantage of intestinal specificity to avoid adverse effects in other organs. In addition, the inhibitory effect of CS‐PLGA nanosystem on intestinal SOAT2 in vivo can be maintained over 72 h.

Our study demonstrated the mechanism on how intestinal SOAT2 being involved in regulation fatty acid absorption. Considering that the SOAT2 activity in gut could be ten times of that in liver,^[^ ^24^ ^]^ intestinal inhibition of SOAT2 would be of more clinical relevance in regulating lipid uptake. Entero‐inhibition of SOAT2 was potent to limit intestinal fatty acid uptake ascribing to enhanced ubiquitination mediated CD36 degradation. CD36 is one of the main transporters mediating fatty acid uptake in cells. Loss of CD36 in mice was shown to reduce ≈50% of fatty acid absorption.^[^ ^25^, ^26^ ^]^ When SOAT2 was inhibited, ubiquitination occurred at the K469 site of CD36, initiating proteasome mediated protein degradation. Dietary long‐chain fatty acids were also proved to trigger ubiquitination of CD36, promoting its significant decline as early as one hour after diet intake.^[^ ^18^ ^]^ CD36 was shown to be a functional substrate of ERAD mediated by RNF5. We showed the CD36 ubiquitination in SOAT2 knockdown cells was mediated by the E3 ligase RNF5, a process triggered by cholesterol‐dependent ER stress. RNF5 recognizes misfolded proteins and promotes their ubiquitination and proteasome‐dependent degradation.^[^ ^27^, ^28^ ^]^ Since tunicamycin causes the accumulation of unfolded proteins in the ER of cells and induces ER stress, it may increase CD36 accumulation in the ER, thereby enhancing its interaction with RNF5. Besides, activation of ER stress resulted in the recruitment of RNF5 and promoted ubiquitination of CD36, which could be reversed by ER stress inhibitor 4‐PBA. Collectively, these findings establish regulation of CD36 stability by RNF5 in response to ER stress. Our present data also suggested the role of changes in cellular cholesterol esterification in controlling CD36 protein levels. Unlike the FA‐induced ubiquitination site,^[^ ^18^ ^]^ changes in cholesterol homeostasis down‐regulate CD36 protein level by inducing ubiquitination at the K469 site of CD36.

## 4 Conclusion

The *Soat2* siRNA/CS‐PLGA nanoparticle system has the advantages of non‐invasive, low systemic toxicity and higher compliance. Our work shows promising translational relevance of intestinal specific *Soat2* siRNA‐based therapy to prevent obese. Though SOAT2 has been known as a regulator to control cholesterol homeostasis, our data added up evidences for the role and mechanism of SOAT2 on regulating intestinal fatty acid absorption, providing the fact that inhibition of this enzyme might be “a stone to hit two birds”.

## 5 Experimental Section

### Materials

CD36 (Cat. ab133625), IRE1α (Cat. ab37073) and SOAT2 antibody (Cat. ab230210) were obtained from Abcam. Ubiquitin (Cat. 20 326), Flag (Cat. 14 793), GFP (Cat. 2555), PDI (Cat. 2446) and CHOP (Cat. 2895) antibodies were purchased from Cell Signaling Technology. HRD1 (Cat. 13473‐1‐AP), CHIP (Cat. 55430‐1‐AP) were obtained from Proteintech. RNF5 (Cat. A8351) antibody was purchased from ABclonal Technology. Tunicamycin (CAS No.:11089‐65‐9), thapsigargin (CAS No.:67526‐95‐8), brefeldin A (CAS No.:20350‐15‐6), Cycloheximide (CAS No.:66‐81‐9), MG132 (CAS No.:133407‐82‐6), 4‐Phenylbutyric acid (CAS No.:1821‐12‐1) were obtained from MedChemExpress. PLGA‐b‐PEG (Cat.765139) and protamine sulfate salt (Cat. P4020) were purchased from Sigma–Aldich.

### Ethical Permission

All the experimental protocols were approved by the Ethical Committee at Nanjing Medical University (IACUC‐2305033).

### Mice, Treatments, and Diets

Intestine‐specific *Soat2* knockout (*Soat2^I‐KO^*) mice were generated by cross‐breeding *Soat2^flox/flox^* mice with *Vil1‐Cre* mice (Figure S12, Supporting Information). Detail procedures were described in Supporting Information.

Adult male *Soat2^I‐KO^* and *Soat2^flox/flox^* mice (age of 7–8 weeks) were given free access to a high fat diet (carbohydrate 44%, fat 42%, of which cholesterol accounted for 0.2%, composed of 4.5 kcal g^−1^ based on caloric content, Tables S2 and S3, Supporting Information) with drinking sugar water (Sucrose 18.9 g L^−1^ and fructose 23.1 g L^−1^, HF/HS diet), or a chow diet for a total 12 weeks (Figure 1A). The body weights of the mice were measured per week. All mice used in the studies were housed in a specific pathogen‐free (SPF) facility with an ambient temperature of 24 ± 1 °C and relative humidity of 40–50% on a 12 h light/dark cycle. After 12 weeks, the mice underwent metabolic cage test.

In order to detect the therapeutic effect of *Soat2* siRNA/CS‐PLGA NPs and the alleviating effect on HFD‐induced obesity of *Soat2* knockdown, adult male mice (age of 7–8 weeks) were given diet supplemented with high fat only. *Soat2* expression was knocked down in the mouse small intestine by garaging CS‐PLGA NPs carrying either a *Soat2* siRNA (at a dose of 40 µg kg^−1^ body weight once every 3 days) or its corresponding control (NC siRNA) for a total of 2 weeks. The following day after final gavage, mice were analyzed and later sacrificed to collet serum, tissues, and feces for further analysis.

### Biochemical Analysis

Plasma alanine aminotransferase (ALT) (Cayman Chemical, Ann Arbor, MI) and aspartate aminotransferase (AST) (BioVision, Milpitas, CA) levels were determined using their enzyme detection kits according to manufacturer's instructions. Triglyceride and cholesterol contents in plasma were measured using commercially available colorimetric kits (Cat#TR22421, Cat#TR13421, Thermo Scientific, Waltham, MA), respectively.

### Intestinal Fatty Acids Absorption

Triolein absorption in mice were examined as previously described^[^ ^29^ ^]^ with modification. Briefly, after 3‐day high fat diet for accommodation, mice were fasted for 10 h and gavaged with 200 µL of olive oil containing 5 µL [9,10‐^3^H(N)]‐triolein (NET431001MC, PerkinElmer, Waltham, MA, USA) during light phase. Small intestine was harvested after 120 min. The small intestine was excised and flushed by PBS with 0.5 mm sodium taurocholate and cut into nine segments with a length of 3 cm. The tissue was chopped and dissolved in tissue solutizer (Biosol, National Diagnostic, Atlanta, GA, USA). The sample was then decolorized with 30% hydrogen peroxide and mixed with scintillation solution (Bioscint, National Diagnostic, Atlanta, GA, USA). Liquid scintillation counters (LS 6500, Beckman Coulter, Brea, CA, USA) were used to measure the ^3^H content.

### Synthesis and Characterizations of CS‐PLGA NPs

CS‐PLGA NPs were synthesized using double‐emulsion and ionic gelation strategies. First, the PLGA NPs were prepared by nanoprecipitation method. In brief, 20 mg of PLGA‐b‐PEG (Sigma‐Aldich, America) polymer was dissolved in 2 mL of methylene dichloride to form a primary emulsion, and a water‐in‐oil emulsion was formed using a probe sonar for 2 min (Ampl 40%, pulse on 10 s, pulse off 5 s) over an ice bath. The primary emulsion was further emulsified in an aqueous polyvinyl alcohol (PVA, Sigma–Aldich, America) solution (10 mL, 1% w/v), and ultrasonicated in an ice bath for 6 min (Ampl 40%, pulse on for 20 s, pulse off for 10 s). Then, the solution was stirred for 3 h to vaporize the solvent. The NPs suspension was then rinsed three times by centrifugation (15 700 rpm, 4 °C, 30 min) and dispersed in double distilled water. The PLGA NPs were used fresh or kept at −80 °C after freeze‐dried for various studies. Second, a stable self‐assembly method was used to prepare CS‐PLGA NPs. A certain amount of PLGA NPs were suspended in 0.4% (w/v) sodium tripolyphosphate solution, slowly added to 1% (w/v) CS solution, and continuously stirred at room temperature for 1 h. The suspension was then centrifuged at 4 °C at 14 000 rpm for 15 min to remove the supernatant. It was then washed twice with double distilled water, and the centrifugally recovered NPs were resuspended in buffer for further use. CS‐PLGA NPs containing *Soat2* siRNA were synthesized using the above method. siRNA entrapment efficiency was the ratio of the RNA contents to the amount of NPs encapsulation. The mean particle size and zeta potential of prepared NPs with or without CS coating were determined using Malvern Zetasizer. The size and morphology of the nano‐carriers were observed by transmission electron microscope.

### Nanocarrier Stability

In order to evaluate the stability of nanocarrier over time under physiological conditions, CS‐PLGA NPs were incubated in simulated gastric fluid and simulated intestinal fluid for 5 h, respectively. The change of mean particle size and PDI of the NPs was analyzed for each hour. These properties were tested at room temperature using the Malvern Zetasizer system.

### Mechanism of Cellular Uptake of CS‐PLGA Nanoparticles

Caco2 cells were seeded in confocal dishes. Cy5‐siRNA NPs (PLGA NPs and CS‐PLGA NPs, respectively) were added to the cells and incubated for 6 h. The cells were then washed to remove the uninternalized particles, fixed with 4% formaldehyde. Cell membranes were labeled with wheat germ agglutinin (WGA). The nuclei were stained with 4′,6‐diamidino‐2‐phenylindole (DAPI). The cellular uptake was observed with Nikon fluorescence microscope.

### Cell Lines and Transfection

Caco2, a cell line derived from a human colon adenocarcinoma, were obtained from National Collection of Authenticated Cell Cultures, China, and were cultured in MEM (Invitrogen) media supplemented with 20% fetal bovine serum (FBS), glutamax, non‐essential amino acids and sodium pyruvate 100 mM solution in a humidified incubator with 5% CO_2_ at 37 °C. The cell line has been tested for mycoplasma contamination. In order to establish a cellular model of high fat diet, cells were monitored until a confluent monolayer was reached and treated for 24 h with a mixture of free fatty acids (FFA, 0.5 mM) including oleate and palmitate in a final ratio of 2:1.

To establish stable cell lines, the PLKO.1‐GFP lentiviral expressing shSOAT2 and negative control were introduced into Caco2 cells. 48–72 h after transfection, stable single clones were selected by sequential dilution. The knockdown efficiency of SOAT2 in the clones was validated by western blot.

### Cellular Fatty Acids Absorption

As previously mentioned, fresh crypts and villi were isolated from the intestine. Cellular fatty acids uptake was analyzed using BODIPY^TM^ FL C_16_ (Thermo Scientific, Waltham, MA) with methods as described.^[^ ^30^ ^]^ The tissues were incubated with 1 µm BODIPY FL C_16_ at 4 °C for 30 min. Trypan blue staining was used to verify cell viability. After incubation, the cells were washed twice with PBS. 4% paraformaldehyde was fixed for 20 min and DAPI was stained for 5 min at room temperature. The intact villi and crypts were observed under Zeiss Confocal laser microscope LSM700.

After treatment with FFA for 2 h, the cells were incubated with 1 µM BODIPY^TM^ FL C_16_ at 37 °C for 30 min. The cells were fixed with 4% paraformaldehyde for 15 min, Nuclei were stained with DAPI for 2 min. Observation was under Zeiss Confocal laser microscope LSM700.

### Cellular Cholesterol Esterification Assay

Cellular cholesterol esterification was analyzed using NBD‐cholesterol (Thermo Scientific, Waltham, MA) with method as described^[^ ^31^ ^]^ with modification. Caco2 cells were seeded onto 35 mm culture dishes and allowed to recover overnight. Assays were done with cells at least 60% confluent. Cells were incubated in medium containing 1 µg mL^−1^ NBD‐cholesterol ethanol solution for 2 h. NBD‐cholesterol was dissolved in ethanol as 1 mg mL^−1^ stock solution (ethanol concentrations did not exceed 0.1%). To prepare microscopic cells, dishes were washed with PBS for three times and fixed with 1 mL 4% paraformaldehyde at room temperature for 20 min. After DAPI staining, cells were examined by Zeiss Confocal laser microscope LSM700 with green channel filters (488 nm excitation, 535 nm emission).

### Immunoprecipitation Assay

After 24 h exposure to FFA, Caco2 cells were treated with 10 µm MG132 for 6 h. The cells were then lysed with a cell lysis/binding buffer (50 mm Tris HCI pH 7.4, 150 mm NaCl, 1 mM EDTA, 1% Triton X. 100). Cell extracts were rotated at 4 °C by anti‐Ras Magnetic Bead Conjugate Antibody (Millipore). After washing with cell lysis/binding buffer for three times, the immune complexes were eluted and performed by western blot.

### Ubiquitination Assay

In the ubiquitination assay, the indicator plasmids were co‐transfected into the Caco2 cells. After 48 h, the cells were treated with FFA for 18 h and then treated with a 10 mm proteasome inhibitor MG132 for another 6 h before harvest. The cells were lysed with lysis/binding buffer and freshly added Proteinase Inhibitor Cocktail (Thermo Scientific, Waltham, MA). The obtained lysates were measured by IP and WB.

### Immunofluorescence Staining of Cells

For anti‐CD36 immunofluorescent staining, cells were fixed in 4% paraformaldehyde for 15mins, and then were hydrated and incubated with anti‐CD36 antibody (1:200) and goat anti‐Rabbit IgG (H+L) cross‐adsorbed secondary antibody, Cy3 (1:500) to evaluate the expression of CD36 in cells. Cell nuclei were stained with 4′,6‐diamidino‐2‐phenylindole (DAPI). Staining sections were examined by Zeiss Confocal laser microscope LSM700.

### Statistical Analysis

All data were expressed as means ± standard error of means (SEM). The data were analyzed by ANOVA. *P* < 0.05 was considered statistically significant. ANOVA follow by Bonferroni test was used to find the difference between two groups. All analysis were performed using SPSS 20.0 software.

## Conflict of Interest

The authors declare no conflict of interest.

## Supporting information



Supporting Information

## Data Availability

The data that support the findings of this study are available in the supplementary material of this article.
