# Supplementary material for: siRNA/CS‐PLGA Nanoparticle System Targeting Knockdown Intestinal SOAT2 Reduced Intestinal Lipid Uptake and Alleviated Obesity
Source: Adv Sci (Weinh). 2024 Sep 19;11(40):2403442. doi: 10.1002/advs.202403442 (PMC11516059; doi:10.1002/advs.202403442)
Supplement: Supplementary file 1 — Supporting Information [file ADVS-11-2403442-s001.docx]

**siRNA/CS-PLGA nanoparticle system targeting knockdown intestinal SOAT2 reduced intestinal lipid uptake and alleviated obesity**

Jingjia Liang^a,c,d, †^, Wentao Shao^b,f, †^, Pu Ni^a,c,d, †^, Qian Liu^a,c,d^, Weirui Kong^a,c,d^, Weiyi Shen^b^, Qihan Wang^b^, Anhua Huang^b^, Guixin Zhang^e^, Yulong Yang^b^, Hongliang Xin^g^, Zhaoyan Jiang^b, *^, Aihua Gu^a,c,d, *^

**Content**

**Materials and Methods**

**Figure S1.** Fat mass of *Soat2^I-KO^* mice fed HF/HS diet measured by CT scanning.

**Figure S2**. Comparison of adipose tissues from *Soat2^I-KO^* and *Soat2^flox^* mice fed with HF/HS diet.

**Figure S3**. Comparison of food intake between *Soat2^I-KO^* and *Soat2^flox^* mice fed with HF/HS diet.

**Figure S4**. Triglyceride (A) and total cholesterol (B) levels in the proximal intestine from *Soat2^I-KO^* and *Soat2^flox^* mice fed with HF/HS diet.

**Figure S5**. The integrity of the proximal small intestine in *Soat2^I-KO^* mice and *Soat2^flox^* mice fed with HF/HS diet.

**Figure S6**. mRNA and protein levels of key transporters related to lipid uptake in intestinal from *Soat2^I-KO^* mice and *Soat2^flox^* mice fed with HF/HS diet.

**Figure S7**. The expression of Soat2 in mice treated with Soat2 siRNA/CS-PLGA nanoparticles.

**Figure S8**. Prediction of ubiquitination site and E3 ligase between CD36 and E3 ligase.

**Figure S9**. Expression of proteins involved in endoplasmic reticulum stress in the proximal intestine of *Soat2^I-KO^* mice and *Soat2^flox^* mice fed with HF/HS diet.

**Figure S10**. The protein level of ER stress in cells treated with FFA or shSOAT2 alone.

**Figure S11**. RNF5-dependent downregulation of CD36 in SOAT2 knockdown cells in associated with cholesterol-dependent protein misfolding.

**Figure S12**. Construction and genotyping of intestine-specific knockout mice.

**Table S1**. The nutrition facts in high fat diet.

**Table S2**. The calorific density in high fat diet.

**Table S3**. Primers used for RT-PCR analyses of the present study.

**Materials and Methods**

**Generation of intestine-specific *Soat*2 knockout (*Soat2^I-KO^*) mice**

*Soat2^flox/flox^* mice were generated by homologous recombination and uses ES cell targeting to modify the *Soat2* gene with *flox* (Supplemental Figure S12C). Briefly, a targeting vector which contains 5.1kb 5' homology arm, 0.8kb flox region, PGK-Neo-polyA, exons 6, 3.6kb 3'homology arm and MC1-TK-polyA negative Screening markers was constructed^1^. The targeting vectors were linearized and electroporated into JAM8A3 ES cells. After screening, a total of 144 G418 and Ganc resistant clones were obtained. Clones were analyzed for correct integration by long-fragment PCR. Correctly targeted clones were microinjected into C57BL/6 blastocysts to obtain chimeric mice. Chimeric mice were mated with Flp mice to obtain positive de-Neo *Soat2^flox/flox^* mice. Intestine -specific *Soat2* knockout (*Soat2^I-KO^*) mice were generated by cross-breeding *Soat2^flox/flox^* mice with *Vil1-Cre* mice. Genotyping of mice harboring the *Soat2^flox^* allele was performed with F1: 5’- TCGTCCCAGCCCAGTCTTT-3’ and R2: 5’- CTGCCTTGCCCACAGTTTCT -3’ resulting in an amplicon of 289 bp of wild-type allele and 344 bp of knockout allele (Supplemental Figure S12D). Genotyping of mice expressing *Vil1-Cre* was performed with primers *Vil1-Cre*-F1: 5’-TCGATGCAACGAGTGATGAG-3’, *Vil1-Cre*-R1 :5’- TCCATGAGTGAACGAACCTG -3’, Control-F1 :5’- CAAATGTTGCTTGTCTGGTG -3’ and Control-R1 :5’- GTCAGTCGAGTGCACAGTTT -3’ resulting in an ~400 bp of target product and an ~200bp of control product (Supplemental Figure S12E).

**Histopathological examination**

Tissues were immobilized overnight in 10% formalin and embedded in paraffin. After dewaxing and rehydration, paraffin sections (5 μm) were prepared with hematoxylin and eosin (H&E, Beyotime Biotechnology Institute, China).

**Oil red o staining**

Frozen sections of intestine with a thickness of 5 μm were fixed and stained with oil red (Sbjbio life science, China) for 15 min. The nuclei were stained with hematoxylin for 2 min. The sections were imaged with light microscopy.

**Assay of triglycerides and cholesterol levels in cells**

Cellular TG levels were determined using kit according to the manufacturer's protocol (Applygen Technologies Inc., Beijing, China) and were normalized to protein concentration and determined by BCA protein Detection kit (Beyotime Biotechnology Institute, China).

**Cell isolation of intestinal epithelium, villi and crypts**

Fresh mouse intestines were rinsed with cold PBS, opened lengthwise, cut into 1 cm slices, and then rotated in 3 mm EDTA at 4 °C PBS. The tissue was then vigorously shaken so that the epithelium was released from the lower muscle tissue. The supernatant was collected as the whole epithelial part and filtered with 70-micron cell filter: intestinal villi on the filter, intestinal crypt through the filter. The cells were centrifuged at 200 g at 4 °C to form balls and then washed with cold PBS. Cells were used for fatty acid uptake, which will be described in a later section.

**Western blot analysis**

Mouse intestine or cultured Caco2 cells were collected and lysed for Western blot analysis. The lysates of intestine tissue and cells were isolated by 8 – 12 % SDS polyacrylamide gel electrophoresis and then electro-transferred to polyvinylidene fluoride membrane. The protein blots were probed with antibodies against CD36 (Abcam) and SOAT2 (abcam). Appropriate secondary antibodies (Cell Signaling Technology, CST) were incubated and the immune response bands were visualized using enhanced chemiluminescence reagents (Bio-RAD, Hercules, CA). Image Lab analysis software was used to analyze the amount of protein, and normalization was performed against their respective controls.

**RNA extraction and real-time PCR**

Total RNA was isolated from cells using TRIzol reagent (Invitrogen, USA) according to manufacturer's instructions. Prime Script RT kit (Takara, China) was used to synthesize cDNA. An LightCycler 480 real-time PCR system was used to perform real-time quantitative PCR on three repeated samples from SYBR Green (Takara, China) reaction mixture. The mRNA levels were standardized with GAPDH and quantified using 2^−ΔΔCT^. Primer sequences are listed in Table S3. Each experiment was conducted at least three times.


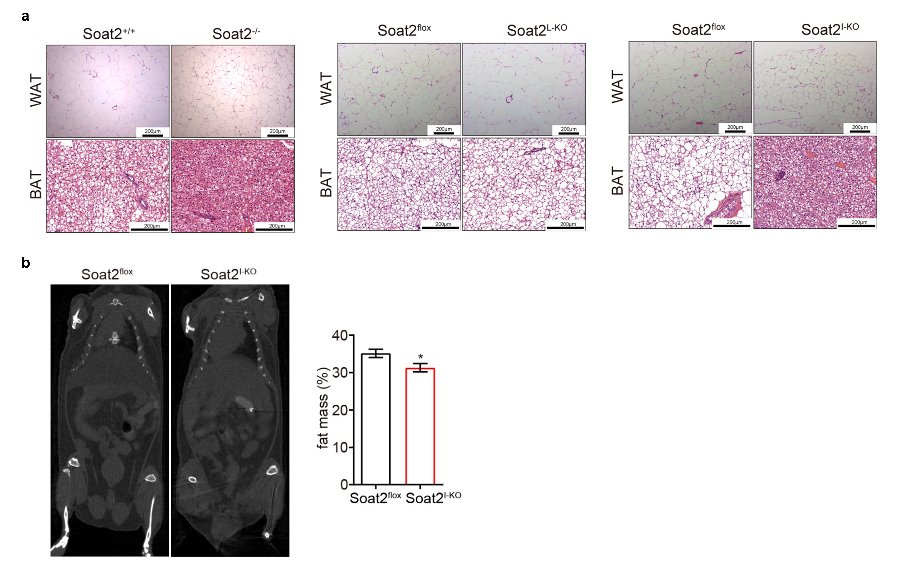


**Figure S1**. Fat mass of *Soat2^I-KO^* mice fed HF/HS diet measured by CT scanning.


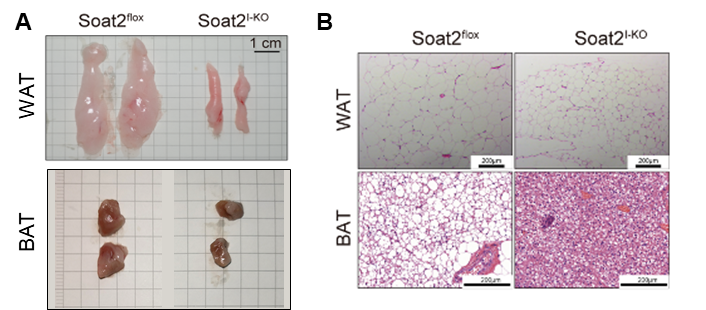


**Figure S2**. Comparison of adipose tissues from *Soat2^I-KO^* and *Soat2^flox^* mice fed with HF/HS diet.


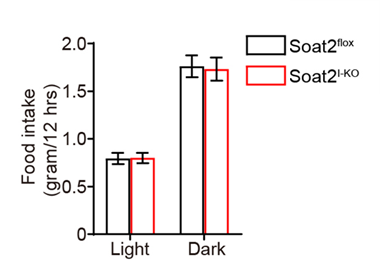


**Figure S3**. Comparison of food intake between *Soat2^I-KO^* and *Soat2^flox^* mice fed with HF/HS diet.


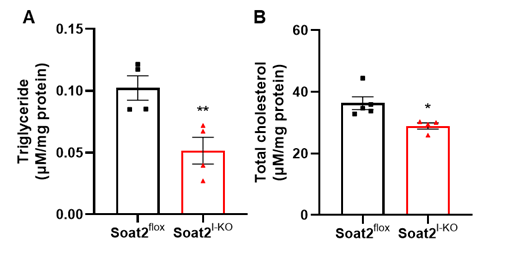


**Figure S4**. Triglyceride (A) and total cholesterol (B) levels in the proximal intestine from *Soat2^I-KO^* and *Soat2^flox^* mice fed with HF/HS diet.


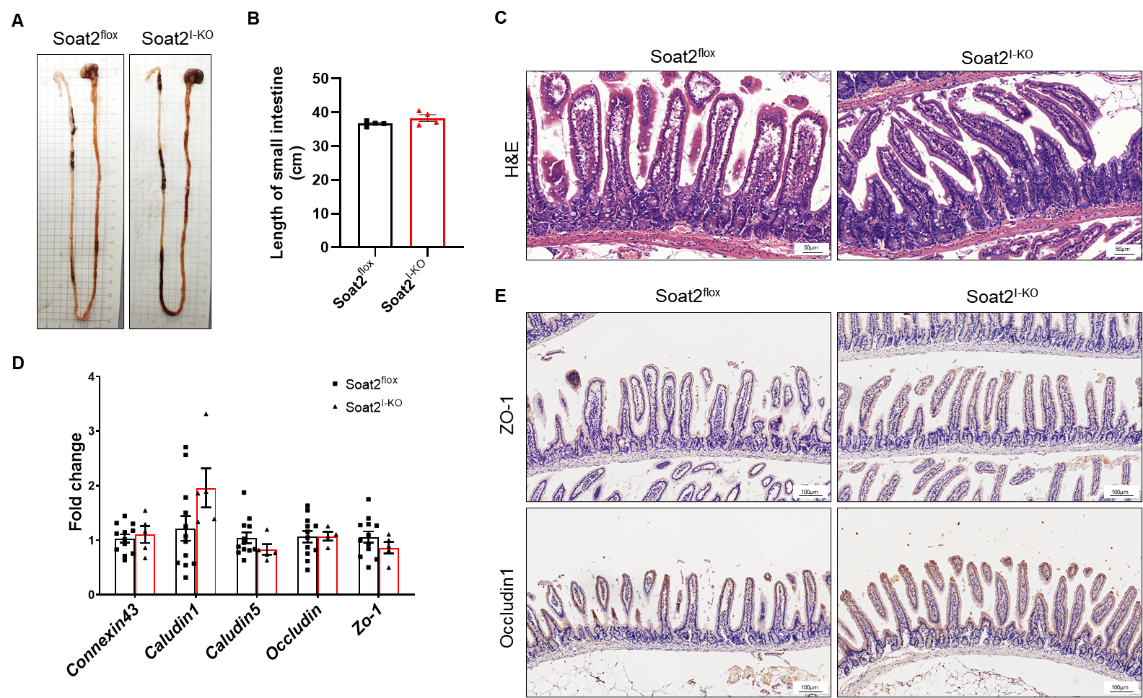


**Figure S5.** The integrity of the proximal small intestine in *Soat2^I-KO^* mice and *Soat2^flox^* mice fed with HF/HS diet.

(A, B) The length of the whole intestine. (C) H&E staining of intestine. (D) The mRNA expression of tight junction proteins in intestine. (E) Immunohistochemical staining of ZO-1 and Occludin1 in intestine.


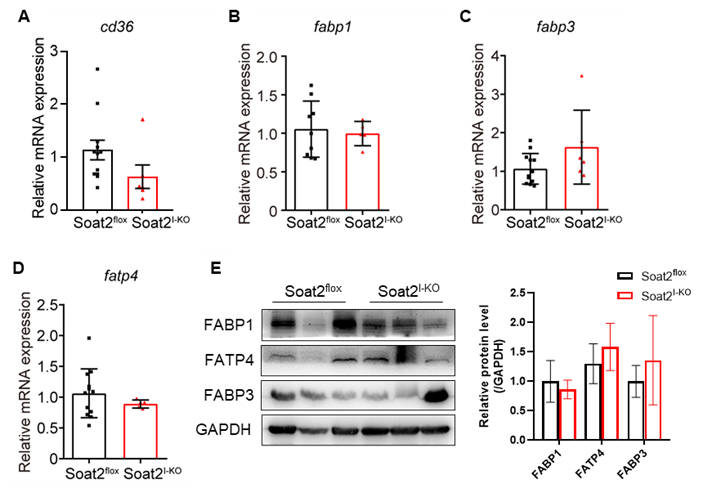


**Figure S6**. mRNA and protein levels of key transporters related to lipid uptake in intestinal from *Soat2^I-KO^* mice and *Soat2^flox^* mice fed with HF/HS diet.

(A, B, C, D) The mRNA expression of genes related to lipid uptake and transportation. (E) The level of proteins related to lipid uptake and transportation.


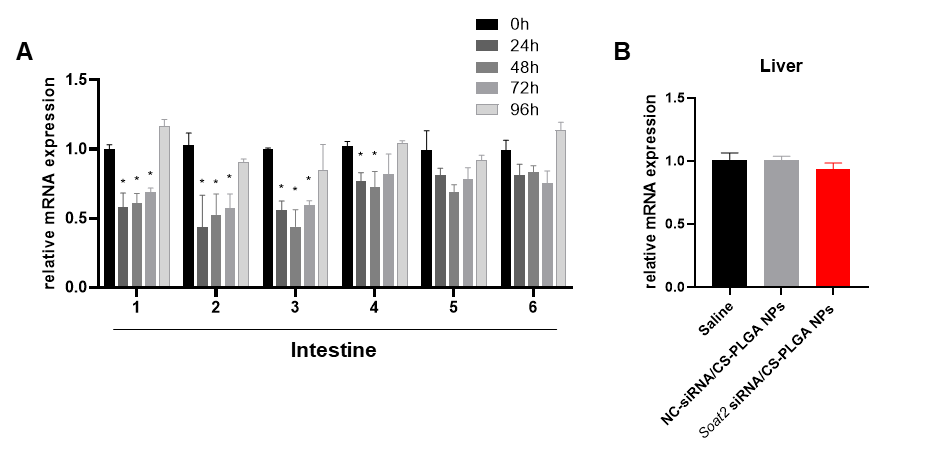


**Figure S7**. The expression of *Soat2* in mice treated with *Soat2* siRNA/CS-PLGA nanoparticles.

(A) Expression of SOAT2 mRNA in each intestinal segment of mice treated with Soat2 siRNA/CS-PLGA NP at each time point (0 h was set as the reference point). (B) The expression of *Soat2* in the liver of mice treated with *Soat2* siRNA/CS-PLGA nanoparticles for 72 h.


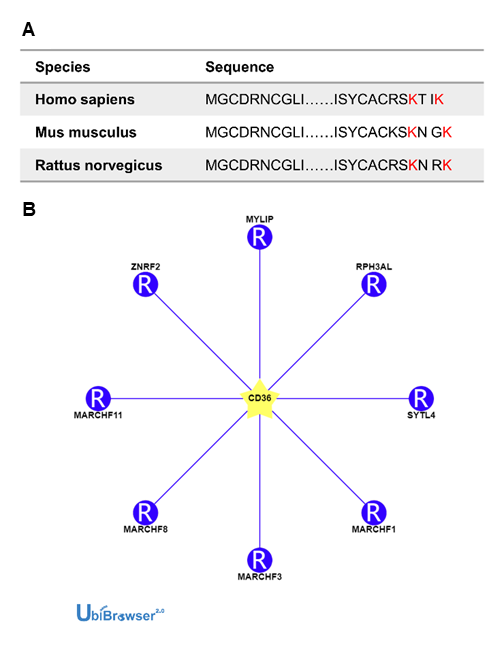


**Figure S8**. Prediction of ubiquitination site and E3 ligase between CD36 and E3 ligase.

(A) Evolutionary conservation analysis revealed that the ubiquitination sites of CD36 is conserved from rat to humans. (B) Predicted E3 ligases associated with CD36 protein ubiquitination on the UbiBrowser website.


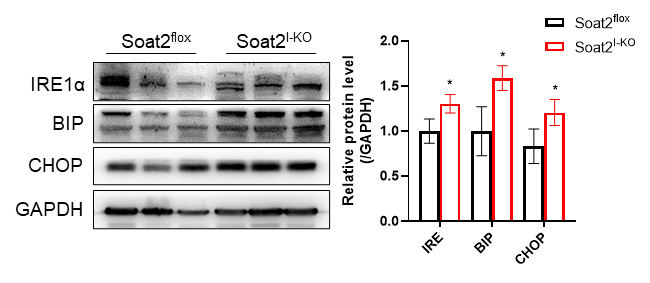


**Figure S9**. Expression of proteins involved in endoplasmic reticulum stress in the proximal intestine of *Soat2^I-KO^* mice and *Soat2^flox^* mice fed with HF/HS diet.


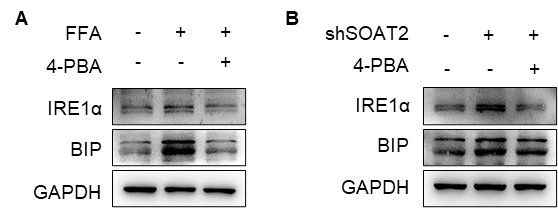


**Figure S10**. The protein level of ER stress in cells treated with FFA (A) or shSOAT2 (B) alone.


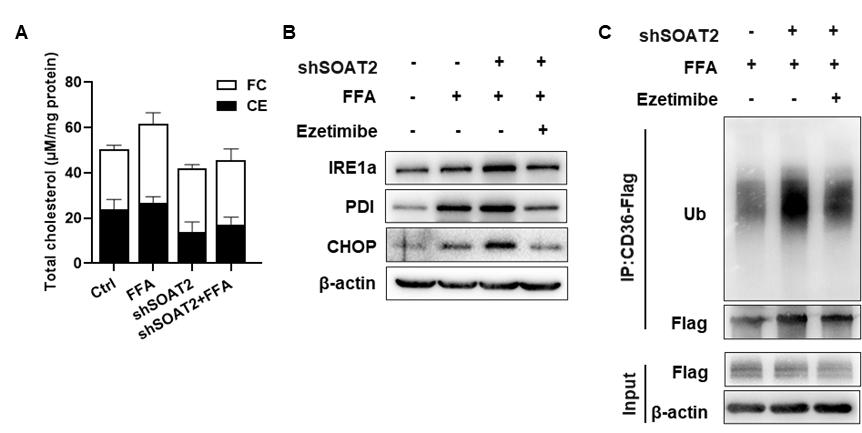


**Figure S11**. RNF5-dependent downregulation of CD36 in SOAT2 knockdown cells in associated with cholesterol-dependent protein misfolding. (A) Cellular free cholesterol and cholesteryl ester levels. (B) Expression of endoplasmic reticulum stress-related proteins in the shSOAT2 Caco2 cells after ezetimibe treatment. (C) The CD36 ubiquitination was determined by IP/western blot after ezetimibe treatment in the presence of MG132 in the shSOAT2 Caco2 cells.


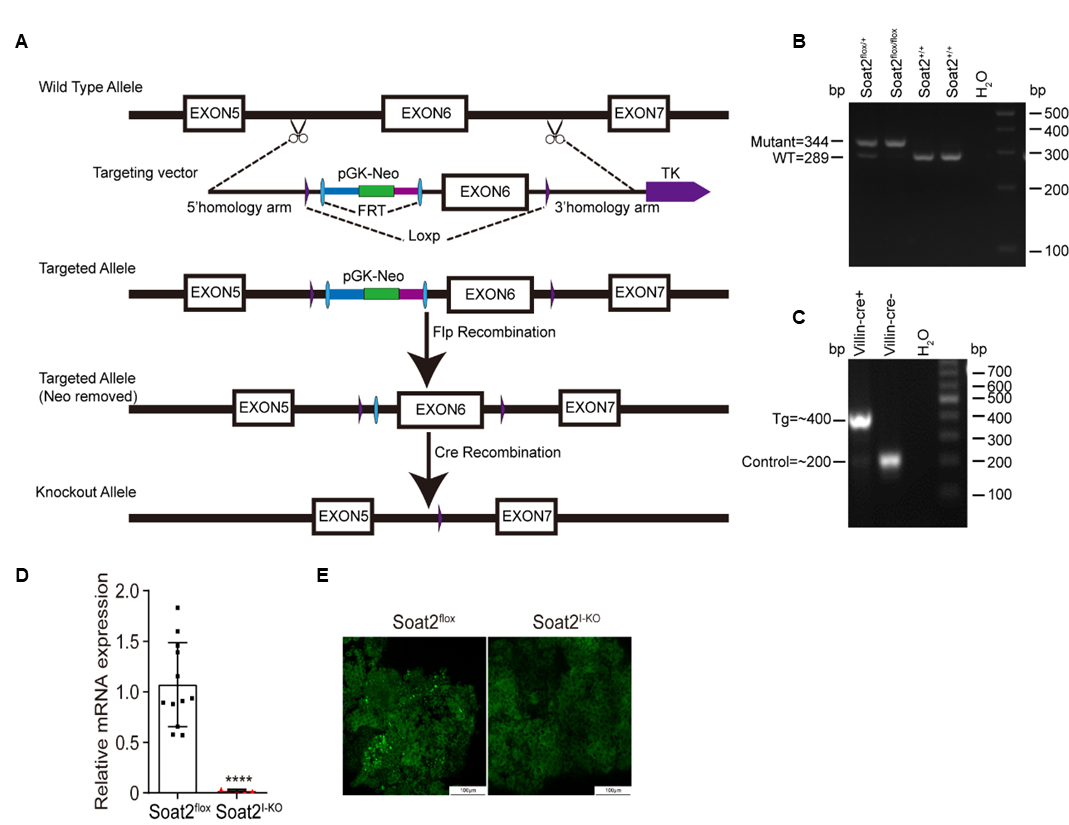


**Figure S12**. Construction and genotyping of intestine-specific knockout mice. (A) Strategy to construct intestine-specific knockout mice. (B, C) Genotyping of mice. (D) Decreased mRNA expression of Soat2 in the small intestine from *Soat2^I-KO^* mice. (E) Decreased SOAT2 activity in small intestine from *Soat2^I-KO^* mice determined by NBD-cholesterol assay.

| **Table S1**. The nutrition facts in high fat diet. | |
| --- | --- |
| Nutrition facts | Quality (g) |
| Protein (casein, L-cystine) | 178.0 |
| Digestible carbohydrates (starch, sucrose) | 489.2 |
| Fats (soybean oil, cream) | 208.8 |
| cellulose | 62.8 |
| vitamins, minerals | 56.5 |
| Choline chloride | 3.1 |
| Cholesterol | 1.5 |
| Antioxidant (TBHQ) | 0.04 |
| Total | 1000 |

| **Table S2**. The calorific density in high fat diet. | |
| --- | --- |
| Calorific density, total 4.5 kcal/g | Percentage |
| Among them, proteins | 14% |
| carbohydrates | 44% |
| Fat | 42% |
| Total | 100% |
| Cholesterol content of about 0.2% | |

| **Table S3** Primers used for RT-PCR analyses in the present study. | | | |
| --- | --- | --- | --- |
| Target | | Forward Primer (5’-3') | Reverse Primer (5’-3') |
| Mouse |  |  |  |
|  | Cd36 | ATGGGCTGTGATCGGAACTG | GTCTTCCCAATAAGCATGTCTCC |
|  | Soat2 | ACAAGACAGACCTCTTCCCTC | ATGGTTCGGAAATGTTGCACC |
|  | Fabp1 | ATGAACTTCTCCGGCAAGTACC | CTGACACCCCCTTGATGTCC |
|  | Fabp3 | ACCTGGAAGCTAGTGGACAG | TGATGGTAGTAGGCTTGGTCAT |
|  | Fatp1 | CGCTTTCTGCGTATCGTCTG | GATGCACGGGATCGTGTCT |
|  | Fatp4 | ACTGTTCTCCAAGCTAGTGCT | GATGAAGACCCGGATGAAACG |
|  | Mttp | CTCTTGGCAGTGCTTTTTCTCT | GAGCTTGTATAGCCGCTCATT |
|  | Gapdh | AGGTCGGTGTGAACGGATTTG | TGTAGACCATGTAGTTGAGGTCA |
| Human |  |  |  |
|  | CD36 | CTTTGGCTTAATGAGACTGGGAC | GCAACAAACATCACCACACCA |
|  | SOAT2 | ATGGAAACACTGAGACGCACA | ATGGAAACACTGAGACGCACA |
|  | GAPDH | ACAACTTTGGTATCGTGGAAGG | GCCATCACGCCACAGTTTC |

**Reference**

1. Chan, W.; Costantino, N.; Li, R.; Lee, S. C.; Su, Q.; Melvin, D.; Court, D. L.; Liu, P., A recombineering based approach for high-throughput conditional knockout targeting vector construction. *Nucleic acids research* **2007,** *35* (8), e64.
